# Effects of semaglutide in obesity‐related heart failure with preserved ejection fraction across the age spectrum: Findings from the STEP‐HFpEF programme

**DOI:** 10.1002/ejhf.70049

**Published:** 2025-11-25

**Authors:** Ambarish Pandey, Michael Moroney, Subodh Verma, Barry A. Borlaug, Javed Butler, Melanie J. Davies, Dalane W. Kitzman, Sanjiv J. Shah, Mark C. Petrie, Cecilia Rönnbäck, Anne Domdey, Søren Rasmussen, Khaja M. Chinnakondepalli, Shachi Patel, Mikhail N. Kosiborod

**Affiliations:** ^1^ Divisions of Cardiology and Geriatrics UT Southwestern Medical Center Dallas TX USA; ^2^ Division of Cardiac Surgery St. Michael's Hospital of Unity Health Toronto Toronto ON Canada; ^3^ School of Medicine Royal College of Surgeons in Ireland Dublin Ireland; ^4^ Division of Cardiac Surgery, Li Ka Shing Knowledge Institute of St Michael's Hospital, Unity Health Toronto University of Toronto Toronto ON Canada; ^5^ Department of Cardiovascular Medicine Mayo Clinic Rochester MN USA; ^6^ Baylor Scott and White Research Institute Dallas TX USA; ^7^ University of Mississippi Jackson MS USA; ^8^ Diabetes Research Centre University of Leicester Leicester UK; ^9^ NIHR Leicester Biomedical Research Centre Leicester UK; ^10^ Department of Cardiovascular Medicine and Section on Geriatrics/Gerontology Wake Forest University School of Medicine Winston‐Salem NC USA; ^11^ Division of Cardiology, Department of Medicine Northwestern University Feinberg School of Medicine Chicago IL USA; ^12^ School of Cardiovascular & Metabolic Health University of Glasgow Glasgow UK; ^13^ Novo Nordisk A/S Søborg Denmark; ^14^ Department of Cardiovascular Disease Saint Luke's Mid America Heart Institute, University of Missouri–Kansas City School of Medicine Kansas MO USA

## Abstract

**Background:**

The prevalence of heart failure with preserved ejection fraction (HFpEF) increases with age, and older adults with HFpEF have worse physical function, quality of life, and clinical outcomes. Semaglutide demonstrated efficacy in the treatment of obesity‐related HFpEF in the STEP‐HFpEF trials. Some have speculated that older patients may have less to gain from incretin therapies (and perhaps more to lose) than younger patients.

**Aims:**

In this pre‐specified pooled subanalysis of the STEP‐HFpEF trials, we evaluated the efficacy of semaglutide across the age spectrum.

**Methods:**

The STEP‐HFpEF and STEP‐HFpEF DM trials enrolled participants with obesity‐related HFpEF and randomized them to semaglutide 2.4 mg once weekly (*n* = 573) or placebo (*n* = 572) for 52 weeks. Dual primary outcomes (change in Kansas City Cardiomyopathy Questionnaire clinical summary score [KCCQ‐CSS] and change in body weight) and secondary outcome measures (6‐minute walk distance [6MWD], C‐reactive protein, hierarchical composite endpoint containing all‐cause death, heart failure events, changes in KCCQ‐CSS and 6MWD) were compared across specific age groups; <55 years, 55–64 years, 65–74 years and ≥75 years.

**Results:**

Among 1145 randomized participants, 8.8% (*N* = 101) were <55, 23.3% (*N* = 267) were aged between 55–64, 42.4% (*N* = 485) were between 65–74, and 25.5% (*N* = 292) were 75 years or over. The efficacy of semaglutide on the dual primary endpoints was consistent across the age spectrum, KCCQ‐CSS (*p*‐interaction = 0.80), and body weight (*p*‐interaction = 0.41). Similar benefits were observed for the key secondary endpoints, with no treatment effect heterogeneity across age groups. Moreover, the safety of semaglutide was consistent across age groups.

**Conclusion:**

In patients with HFpEF enrolled across the STEP‐HFpEF and STEP‐HFpEF DM trials, treatment with semaglutide improved disease‐specific symptoms, physical function and reduced body weight across the age spectrum. The safety profile of semaglutide was consistent in older and younger patients.

## Background

Obesity‐related heart failure (HF) with preserved ejection fraction (HFpEF) is characterized by worse quality of life, impaired physical function, and high cardiac filling pressures.^1^, ^2^, ^3^ Older patients with obesity‐related HFpEF have a high burden of sarcopenia and physical dysfunction and are at a higher risk of adverse clinical outcomes.^4^ Incretin‐based weight loss therapies have demonstrated efficacy in improving disease‐specific symptoms, physical function, and exercise capacity among patients with obesity‐related HFpEF.^5^, ^6^ However, their efficacy across the age spectrum in HFpEF is unknown. Additionally, there have been concerns that the use of weight loss therapies in older adults may worsen sarcopenia.^7^, ^8^, ^9^ We performed a pre‐specified, age‐stratified analysis of the pooled participant‐level data from the randomized, double‐blind, placebo‐controlled STEP‐HFpEF programme (STEP‐HFpEF and STEP‐HFpEF DM) to evaluate the efficacy of semaglutide in HFpEF across the age spectrum.

## Methods

The design and primary results of the individual trials and the overall programme have been published previously.^5^, ^6^ Institutional review board or ethics committee approval was obtained at each study site, and all patients provided written informed consent.

### Study population

The inclusion and exclusion criteria for both STEP‐HFpEF trials have been reported previously.^5^, ^6^ Briefly, the trial enrolled participants with symptomatic HF (left ventricular ejection fraction ≥45%) and obesity (body mass index [BMI] ≥30 kg/m^2^) with objective evidence of HFpEF based on elevated filling pressures or elevated natriuretic peptide levels and echocardiographic abnormalities, or HF hospitalization in the previous 12 months. Only patients with type 2 diabetes mellitus (T2DM) were included in the STEP‐HFpEF DM trial, in which semaglutide or placebo was added to background therapy for T2DM. Eligible participants were randomized 1:1 to receive once‐weekly semaglutide 2.4 mg or matching placebo in addition to standard care for 52 weeks.

### Outcomes

The dual primary endpoints were: (1) change in Kansas City Cardiomyopathy Questionnaire clinical summary score (KCCQ‐CSS) and percentage change in body weight from baseline to 52 weeks. The confirmatory secondary endpoints were changes in 6‐min walk distance (6MWD) and C‐reactive protein (CRP), and a hierarchical composite endpoint (comprised of all‐cause death [from baseline to 57 weeks]). HF events (hospitalizations or urgent visits requiring intravenous therapy; an exploratory endpoint in both STEP‐HFpEF trials) were adjudicated by a blinded clinical events committee as previously described.^5^, ^6^ Change in N‐terminal pro‐B‐type natriuretic peptide (NT‐proBNP) concentrations from baseline to week 52 was also evaluated as an exploratory endpoint. Serious adverse events (SAEs), SAEs leading to permanent treatment discontinuation, and cardiac and gastrointestinal SAEs were also evaluated as safety endpoints. Finally, atrial fibrillation events and bone/joint injuries were also assessed as safety endpoints of interest for this analysis.

### Statistical analysis

The study participants were stratified according to age into four categories: (i) <55 years, (ii) 55–64 years, (iii) 65–74 years, and (iv) ≥75 years. Baseline characteristics were compared across age strata using the Wilcoxon test and the Chi‐square test for continuous and categorical variables, respectively. The efficacy endpoints were examined according to the intention‐to‐treat principle. Analyses of continuous endpoints were performed using analysis of covariance models adjusted for the baseline value of the endpoint, treatment arm, trial, and BMI (<35 kg/m^2^ or ≥35 kg/m^2^) as fixed factors using 1000 imputations; analyses also included an interaction term between treatment arm and age category. Estimates were combined using Rubin's rule. For outcomes of change in KCCQ‐CSS and 6MWD, missing observations at week 52 caused by cardiovascular death or previous HF events (if not observed) were single‐imputed using the lowest observed value across both treatment arms and visits. Missing values caused by other reasons were imputed from retrieved participants in the same randomized treatment arm. For other endpoints, missing observations at week 52 were imputed irrespective of death or prior HF events using the same imputation method. Interaction *p*‐values were derived from an F‐test of equality between the treatment differences across the age subgroups.

Analyses of the hierarchical composite endpoint (win ratio) were performed stratified by age, based on direct comparisons of each participant in semaglutide versus placebo arms, as reported previously.^5^, ^6^ A two‐sided *p*‐value of <0.05 was considered statistically significant and no adjustment for multiplicity was performed. Results are presented as estimated changes from baseline to 52 weeks for continuous endpoints and a win ratio for the hierarchical composite endpoint with 95% confidence intervals (CI) and two‐sided *p*‐values. NT‐proBNP and CRP were log‐transformed; hence, the treatment ratio with the corresponding 95% CI is reported. Statistical analyses were performed using SAS version 9.4 (SAS/STAT version 15.1).

## Results

### Baseline characteristics by Age

The median age of participants was 69 years (interquartile range: 62–75). Participants in the older age subgroups (65–74 years and ≥75 years) had lower body weight but a higher prevalence of cardiovascular comorbidities than younger participants. Older participants had higher NT‐proBNP, while CRP was highest among younger participants. Older participants had lower 6MWD and had a larger proportion with New York Heart Association class III symptoms than younger participants. There were no differences in KCCQ‐CSS scores by age category at baseline. No differences were noted in the use of sodium‐glucose co‐transporter‐2 inhibitors and mineralocorticoid receptor antagonists by age category (*Table* *1*).

**Table 1:** Baseline characteristics stratified by age

Characteristic	Age (years)				*p*‐value
	<55 (*n* = 101)	55–64 (*n* = 267)	65–74 (*n* = 485)	≥75 (*n* = 292)	
Female sex	53 (52.5)	119 (44.6)	243 (50.1)	155 (53.1)	0.2138
Race^a^					<0.0001
Asian	16 (15.8)	31 (11.6)	22 (4.5)	7 (2.4)	
Black or African American	8 (7.9)	13 (4.9)	14 (2.9)	4 (1.4)	
White	76 (75.2)	223 (83.5)	446 (92.0)	281 (96.2)	
Other	1 (1.0)	0 (0.0)	3 (0.6)	0 (0.0)	
Diabetes duration, years^b^	6.4 (2.4–13.3)	7.6 (3.8–13.3)	7.5 (3.9–14.9)	10.2 (4.6–17.0)	0.0793
Body weight, kg	117.2 (98.2–141.7)	112.0 (94.2–132.0)	103.5 (93.8–116.8)	96.7 (87.5–107.4)	<0.0001
BMI, kg/m^2^	39.5 (35.5–46.9)	38.4 (34.6–43.6)	36.8 (33.7–41.0)	35.2 (32.5–38.8)	<0.0001
Waist circumference, cm	124.0 (114.3–139.0)	124.0 (114.0–134.0)	120.0 (111.0–128.2)	116.8 (109.2–124.0)	<0.0001
SBP, mmHg	130.0 (117.0–140.0)	134.0 (125.0–144.0)	133.0 (123.0–144.0)	135.5 (123.5–145.0)	0.0086
NYHA class					0.0182
II	75 (74.3)	197 (73.8)	331 (68.2)	182 (62.3)	
III	26 (25.7)	70 (26.2)	154 (31.8)	108 (37.0)	
IV	0 (0.0)	0 (0.0)	0 (0.0)	2 (0.7)	
LVEF, %	55.0 (49.0–60.0)	56.0 (50.0–61.0)	56.0 (50.0–60.0)	58.0 (51.5–60.0)	0.3453
KCCQ‐CSS, points	59.4 (50.0–71.9)	59.6 (43.8–74.0)	59.8 (44.8–72.9)	57.3 (38.8–69.5)	0.0782
6MWD, m	346.2 (262.5–400.0)	321.1 (240.0–380.8)	297.3 (227.7–374.2)	258.5 (183.1–327.5)	<0.0001
CRP, mg/L	7.9 (2.6–15.0)	4.2 (2.1–9.0)	3.4 (1.7–7.4)	2.8 (1.4–6.4)	<0.0001
NT‐proBNP, pg/ml	231.3 (143.8–464.7)	369.6 (186.9–771.3)	500.7 (242.7–983.5)	782.7 (369.1–1376.0)	<0.0001
Medical history					
Hypertension	71 (70.3)	218 (81.6)	420 (86.6)	250 (85.6)	0.0005
Atrial fibrillation	25 (24.8)	95 (35.6)	227 (46.8)	171 (58.6)	<0.0001
OSA	9 (8.9)	31 (11.6)	50 (10.3)	29 (9.9)	0.8654
CAD	18 (17.8)	43 (16.1)	116 (23.9)	69 (23.6)	0.0486
Medications					
Diuretics	78 (77.2)	202 (75.7)	398 (82.1)	247 (84.6)	0.0355
Loop diuretics	61 (60.4)	150 (56.2)	285 (58.8)	206 (70.5)	0.0020
Thiazides	16 (15.8)	32 (12.0)	81 (16.7)	46 (15.8)	0.3813
Beta blockers	79 (78.2)	214 (80.1)	395 (81.4)	240 (82.2)	0.8099
SGLT2 inhibitors	25 (24.8)	58 (21.7)	87 (17.9)	51 (17.5)	0.2469
MRA	40 (39.6)	88 (33.0)	167 (34.4)	89 (30.5)	0.3758
ACE‐I/ARB/ARNI	74 (73.3)	208 (77.9)	387 (79.8)	230 (78.8)	0.5344
ARNI	9 (8.9)	16 (6.0)	20 (4.1)	13 (4.5)	0.1950
Insulin and analogues	13 (12.9)	33 (12.4)	51 (10.5)	31 (10.6)	0.8077
Sulfonylureas	11 (10.9)	36 (13.5)	41 (8.5)	20 (6.8)	0.0421
DPP‐4 inhibitors	12 (11.9)	24 (9.0)	35 (7.2)	21 (7.2)	0.3812

Categorical variables are shown as *n* (%) and continuous variables as median (Q1–Q3).

6MWD, 6‐minute walk distance; ACE‐I, angiotensin‐converting enzyme inhibitor; ARB, angiotensin receptor blocker; ARNI, angiotensin receptor–neprilysin inhibitor; BMI, body mass index; CAD, coronary artery disease; DPP‐4, dipeptidyl peptidase‐4; CRP, C‐reactive protein; KCCQ‐CSS, Kansas City Cardiomyopathy Questionnaire clinical summary score; LVEF, left ventricular ejection fraction; MRA, mineralocorticoid receptor antagonist; NT‐proBNP, N‐terminal pro‐B‐type natriuretic peptide; NYHA, New York Heart Association; OSA, obstructive sleep apnoea; SBP, systolic blood pressure; SGLT2, sodium–glucose co‐transporter‐2.

^a^ Race was reported by the investigator.

^b^ Diabetes was an exclusion criterion in the STEP‐HFpEF trial, therefore, the data shown are from the STEP‐HFpEF DM trial only.

### Efficacy of semaglutide versus placebo by Age

**Figure 1:**
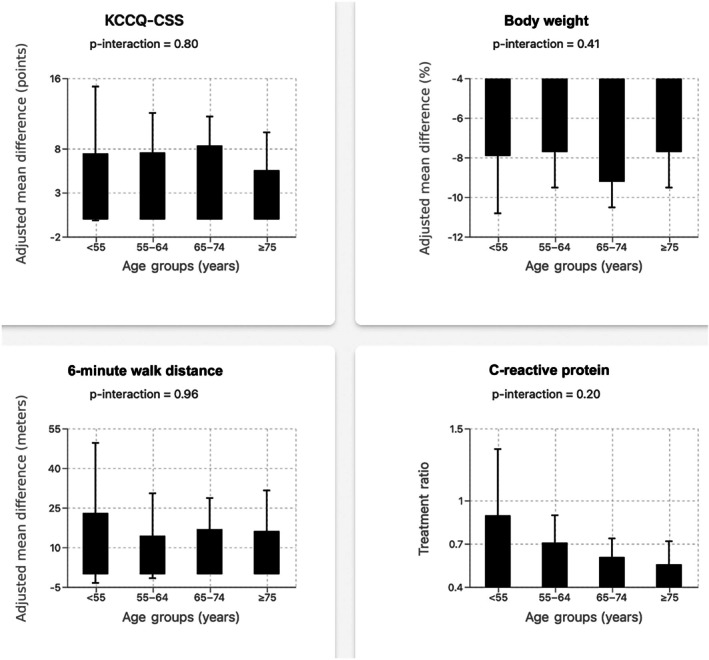
Treatment effect of semaglutide (vs. placebo) on key primary and secondary outcomes among participants across different age strata. The bar plots show the adjusted mean difference between semaglutide (vs. placebo) in change in key outcomes (Kansas City Cardiomyopathy Questionnaire clinical summary score [KCCQ‐CSS], body weight, 6‐minute walk distance) from baseline to 52‐week follow‐up across different age strata. For C‐reactive protein, the plot shows the treatment ratio for semaglutide versus placebo.

The effect of semaglutide on the dual primary endpoint of KCCQ‐CSS and body weight was consistent across age strata without any significant heterogeneity in the treatment effect by age (*Figure* *1*, *Table* *2*). For body weight, a significant reduction ranging from −7.7% to −9.2% was observed across all age groups (*p*‐interaction = 0.41). Similarly, semaglutide use resulted in 5.6‐point to 8.4‐point improvement of KCCQ‐CSS across the age groups (*p*‐interaction = 0.80). Among confirmatory secondary endpoints, semaglutide led to an increase in 6MWD (*p*‐interaction = 0.96) and a favourable win ratio for the hierarchical composite endpoint (*p*‐interaction = 0.85) in each age category without significant heterogeneity in treatment effect. Semaglutide was also associated with consistent reductions in CRP and NT‐proBNP across different age groups (*p*‐interaction: CRP = 0.20, NT‐proBNP = 0.78). In terms of safety, the proportion of participants with SAEs was higher in the older age groups, with a higher frequency noted in the placebo group versus the semaglutide arm (*Table* *3*). Frequency of investigator‐reported atrial fibrillation events and bone/joint injuries on follow‐up was low, with no difference between the semaglutide and placebo arms across age strata (*Table* *3*).

**Table 2:** Effect of semaglutide versus placebo on outcomes by age categories

Outcome^a^	Age (years)								*p*‐interaction
	<55 (*n* = 101)		55–64 (*n* = 267)		65–74 (*n* = 485)		≥75 (*n* = 292)		
	Semaglutide (*n* = 36)	Placebo (*n* = 52)	Semaglutide (*n* = 131)	Placebo (*n* = 110)	Semaglutide (*n* = 240)	Placebo (*n* = 218)	Semaglutide (*n* = 125)	Placebo (*n* = 141)	
Dual primary endpoint									
Change in KCCQ‐CSS at 52 weeks, points	14.9	7.4	16.0	8.4	16.5	8.1	11.3	5.8	
Adjusted mean difference, points	7.5 (−0.1, 15.1)		7.6 (3.1, 12.1)		8.4 (5.0, 11.7)		5.6 (1.2, 9.9)		0.7956
Change in body weight at 52 weeks, %	−10.5	−2.6	−10.2	−2.5	−12.1	−2.9	−11.5	−3.8	
Adjusted mean difference, %	−7.9 (−10.8, −4.9)		−7.7 (−9.5, −5.9)		−9.2 (−10.5, −7.9)		−7.7 (−9.5, −6.0)		0.4127
Confirmatory secondary endpoints									
Change in 6MWD at 52 weeks, m	26.1	3.0	20.8	6.2	18.9	1.8	6.0	−10.3	
Adjusted mean difference, m	23.2 (−3.3, 49.7)		14.5 (−1.5, 30.6)		17.0 (5.2, 28.8)		16.3 (0.9, 31.7)		0.9588
Hierarchical composite endpoint, win ratio	1.56 (0.94, 2.58)		1.82 (1.32, 2.51)		1.73 (1.37, 2.20)		1.43 (1.08, 1.90)		0.8511
CRP ratio at 52 weeks (follow‐up/baseline)	0.76	0.84	0.62	0.88	0.54	0.88	0.53	0.95	
Treatment ratio	0.90 (0.59, 1.36)		0.71 (0.55, 0.90)		0.61 (0.51, 0.74)		0.56 (0.44, 0.72)		0.2036
NT‐proBNP ratio at 52 weeks	0.72	0.81	0.65	0.85	0.79	0.98	0.94	1.07	
Treatment ratio	0.89 (0.62, 1.29)		0.76 (0.61, 0.96)		0.81 (0.69, 0.94)		0.88 (0.71, 1.08)		0.7817

Data for in‐trial period for participants with an observation at week 52. Values in brackets are 95% confidence intervals. *p*‐values are for interaction between treatment × age category.

6MWD, 6‐minute walk distance; CRP, C‐reactive protein; KCCQ‐CSS, Kansas City Cardiomyopathy Questionnaire clinical summary score; NT‐proBNP, N‐terminal pro‐B‐type natriuretic peptide.

^a^ The number of participants for each outcome varies on availability of outcome at week 52 and is listed above each outcome of interest.

**Table 3:** Adverse events across age spectrum among participants across semaglutide versus placebo arms

Outcomes	Age (years)							
	<55 (*n* = 101)		55–64 (*n* = 267)		65–74 (*n* = 485)		≥75 (*n* = 292)	
	Semaglutide	Placebo	Semaglutide	Placebo	Semaglutide	Placebo	Semaglutide	Placebo
Serious adverse events	3 (7.5)	11 (18.0)	27 (19.0)	28 (22.4)	39 (15.3)	65 (28.3)	31 (22.8)	61 (39.1)
Cardiac disorders	1 (2.5)	6 (9.8)	12 (8.5)	14 (11.2)	7 (2.7)	22 (9.6)	12 (8.8)	33 (21.2)
Atrial fibrillation/flutter	0	2 (3.3)	4 (2.8)	2 (1.6)	3 (1.2)	10 (4.3)	3 (2.2)	5 (3.2)
Bone and joint injuries	1 (2.5)	0	0	1 (0.8)	3 (1.2)	2 (0.9)	4 (2.9)	3 (1.9)
Infection and infestations	1 (2.5)	3 (4.9)	9 (6.3)	2 (1.6)	6 (2.4)	20 (8.7)	6 (4.4)	19 (12.2)
Gastrointestinal disorders	0 (0)	0 (0)	4 (2.8)	1 (0.8)	7 (2.7)	5 (2.2)	5 (3.7)	6 (3.8)
Injury, poisoning, and procedural complications	1 (2.5)	0 (0)	2 (1.4)	2 (1.6)	5 (2.0)	4 (1.7)	4 (2.9)	4 (2.6)
Hepatobiliary disorders	1 (2.5)	0 (0)	1 (0.7)	0 (0)	1 (0.4)	1 (0.4)	3 (2.2)	3 (1.9)
Nervous system disorders	1 (2.5)	0 (0)	3 (2.1)	2 (1.6)	6 (2.4)	7 (3.0)	7 (5.1)	5 (3.2)
Renal and urinary disorders	1 (2.5)	2 (3.3)	3 (2.1)	1 (0.8)	3 (1.2)	5 (2.2)	1 (0.7)	5 (3.2)
Metabolism and nutrition disorders	1 (2.5)	0 (0)	4 (2.8)	0 (0)	2 (0.8)	3 (1.3)	0 (0)	5 (3.2)
Neoplasms benign, malignant, and unspecified (including cysts and polyps)	0 (0)	0 (0)	1 (0.7)	1 (0.8)	6 (2.4)	6 (2.6)	4 (2.9)	4 (2.6)
Blood and lymphatic system disorders	0 (0)	0 (0)	1 (0.7)	0 (0)	0 (0)	0 (0)	2 (1.5)	4 (2.6)
Vascular disorders	0 (0)	0 (0)	0 (0)	1 (0.8)	5 (2.0)	7 (3.0)	2 (1.5)	2 (1.3)
Respiratory, thoracic, and mediastinal disorders	0 (0)	1 (1.6)	2 (1.4)	3 (2.4)	3 (1.2)	7 (3.0)	1 (0.7)	7 (4.5)
Reproductive system and breast disorders	0 (0)	0 (0)	0 (0)	0 (0)	1 (0.4)	0 (0)	1 (0.7)	1 (0.6)
General disorders and administration site conditions	0 (0)	1 (1.6)	1 (0.7)	0 (0)	0 (0)	5 (2.2)	1 (0.7)	0 (0)
Musculoskeletal and connective tissue disorders	0 (0)	2 (3.3)	4 (2.8)	3 (2.4)	5 (2.0)	5 (2.2)	0 (0)	2 (1.3)
Eye disorders	0 (0)	0 (0)	0 (0)	1 (0.8)	0 (0)	1 (0.4)	0 (0)	1 (0.6)
Immune system disorders	0 (0)	1 (1.6)	1 (0.7)	1 (0.8)	0 (0)	0 (0)	0 (0)	0 (0)
Ear and labyrinth disorders	0 (0)	0 (0)	0 (0)	0 (0)	0 (0)	2 (0.9)	2 (1.5)	0 (0)
Investigations	0 (0)	0 (0)	1 (0.7)	1 (0.8)	1 (0.4)	0 (0)	0 (0)	1 (0.6)
Congenital, familial, and genetic disorders	0 (0)	0 (0)	0 (0)	1 (0.8)	0 (0)	5 (2.2)	0 (0)	0 (0)
Psychiatric disorders	0 (0)	0 (0)	0 (0)	0 (0)	0 (0)	1 (0.4)	0 (0)	0 (0)
Endocrine disorders	0 (0)	0 (0)	0 (0)	0 (0)	0 (0)	1 (0.4)	0 (0)	0 (0)
Skin and subcutaneous disorders	0 (0)	0 (0)	0 (0)	0 (0)	0 (0)	2 (0.9)	0 (0)	1 (0.6)
Surgical and medical procedures	0 (0)	0 (0)	0 (0)	0 (0)	0 (0)	1 (0.4)	0 (0)	1 (0.6)

Data are presented as *n* (%). Adverse events are from the in‐trial period using the safety analysis set.

## Discussion

**Figure 2:**
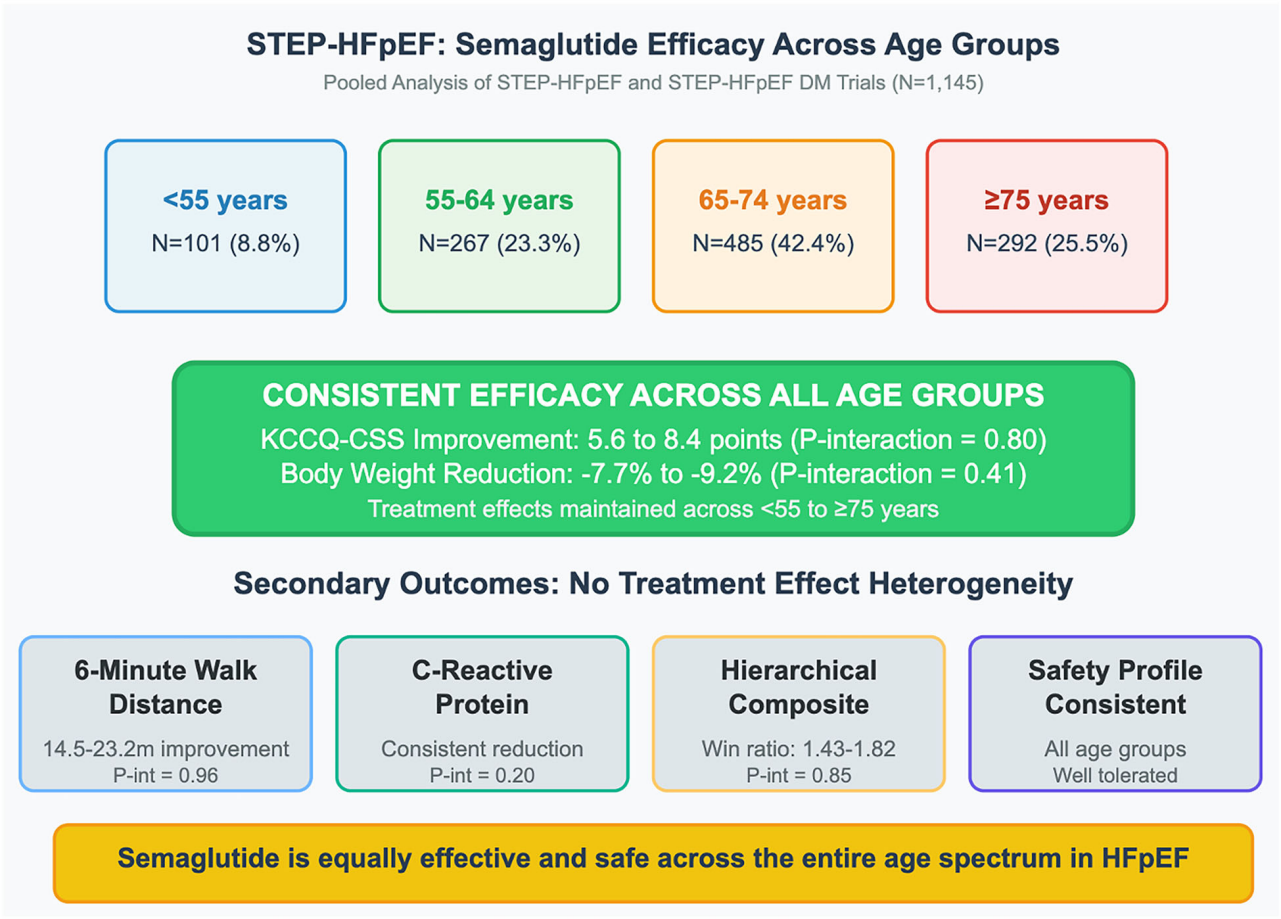
Efficacy and safety of semaglutide across age groups in patients with obesity‐related HFpEF. KCCQ‐CSS, Kansas City Cardiomyopathy Questionnaire clinical summary score; HFpEF, heart failure with preserved ejection fraction.

In this pre‐specified, pooled, patient‐level, secondary analysis of the STEP‐HFpEF programme trials, we observed that older participants with obesity‐related HFpEF had a higher burden of comorbidities, lower functional status, lower exercise capacity, and higher NT‐proBNP levels. In contrast, younger patients had higher body weight and a greater burden of inflammation. The favourable effect of semaglutide was consistent across the age spectrum for the primary and key secondary outcomes with no significant treatment heterogeneity (*Figure* *2*). Moreover, the lower rates of SAEs with semaglutide versus placebo was consistent across age categories.

HFpEF has emerged as a true geriatric syndrome with the highest prevalence among older adults who have sarcopenic obesity and a high burden of comorbidities, sarcopenia, frailty, and physical function impairment.^4^ As a result, there have been concerns about worsening malnutrition in these patients with the use of weight loss agents, like semaglutide, which may potentially worsen frailty and sarcopenia.^7^, ^10^ Our findings help to alleviate these concerns by demonstrating that adults with obesity‐related HFpEF derive similar treatment benefits from semaglutide regardless of age. We observed that semaglutide improved disease‐specific symptoms, physical function, and exercise capacity, even amongst the oldest participants. These findings support the use of semaglutide as an effective therapy for obesity‐related HFpEF, even amongst older patients for whom there may be greater therapeutic inertia due to concerns of side effects, higher frailty burden, and lower BMI. It is noteworthy that we do not have objective measures of lean body mass and frailty available in the study, limiting our ability to assess the effects of semaglutide on these meaningful parameters. This is particularly relevant considering the high burden of frailty in patients with HF, particularly HFpEF.^11^, ^12^ Furthermore, prior studies have demonstrated substantial loss of lean mass with the use of glucagon‐like peptide‐1 receptor agonists (GLP‐1RAs).^8^, ^9^ Future studies with objective assessment of body composition and frailty burden are needed to address these knowledge gaps. While a higher frequency of adverse events, including atrial fibrillation, was noted in older participants, the use of semaglutide was not associated with a meaningful increase in risk of adverse events in any age group, highlighting its safety and tolerability across the age spectrum. Specifically, we did not see increased bone fractures/joint injuries with semaglutide use in older participants, which is reassuring considering the prior reports of loss of bone mineral density with use of this therapy.^13^

Several limitations to our study are noteworthy. First, owing to the trial‐specific inclusion/exclusion criteria, the study population may not be generalizable to the broader population of HFpEF patients, which includes a larger proportion of older patients with a high burden of frailty.^11^ Second, the study is not powered to assess the efficacy of semaglutide on clinical outcomes, and future studies with longer‐term follow‐up are needed to establish the clinical efficacy of GLP‐1RAs in HFpEF, particularly among older patients. In conclusion, semaglutide is effective in treating obesity‐related HFpEF across the age spectrum. The safety profile of semaglutide was consistent across age groups. Future studies are required to characterize its impact on clinical events, long‐term outcomes, and safety.

## References

[1] Borlaug BA , Jensen MD , Kitzman DW , Lam CSP , Obokata M , Rider OJ . Obesity and heart failure with preserved ejection fraction: New insights and pathophysiological targets. Cardiovasc Res 2023;118:3434–3450. 10.1093/cvr/cvac120 35880317 PMC10202444

[2] Kitzman DW , Nicklas BJ . Pivotal role of excess intra‐abdominal adipose in the pathogenesis of metabolic/obese HFpEF. JACC Heart Fail 2018;6:1008–1010. 10.1016/j.jchf.2018.08.007 30316933 PMC7710134

[3] Obokata M , Reddy YNV , Pislaru SV , Melenovsky V , Borlaug BA . Evidence supporting the existence of a distinct obese phenotype of heart failure with preserved ejection fraction. Circulation 2017;136:6–19. 10.1161/CIRCULATIONAHA.116.026807 28381470 PMC5501170

[4] Pandey A , Shah SJ , Butler J , Kellogg DL Jr , Lewis GD , Forman DE , *et al*. Exercise intolerance in older adults with heart failure with preserved ejection fraction: JACC state‐of‐the‐art review. J Am Coll Cardiol 2021;78:1166–1187. 10.1016/j.jacc.2021.07.014 34503685 PMC8525886

[5] Kosiborod MN , Abildstrom SZ , Borlaug BA , Butler J , Rasmussen S , Davies M , *et al*.; STEP‐HFpEF Trial Committees and Investigators . Semaglutide in patients with heart failure with preserved ejection fraction and obesity. N Engl J Med 2023;389:1069–1084. 10.1056/NEJMoa2306963 37622681

[6] Kosiborod MN , Petrie MC , Borlaug BA , Butler J , Davies MJ , Hovingh GK , *et al*.; STEP‐HFpEF DM Trial Committees and Investigators . Semaglutide in patients with obesity‐related heart failure and type 2 diabetes. N Engl J Med 2024;390:1394–1407. 10.1056/NEJMoa2313917 38587233

[7] Driggin E , Goyal P . Malnutrition and sarcopenia as reasons for caution with GLP‐1 receptor agonist use in HFpEF. J Card Fail 2024;30:610–612. 10.1016/j.cardfail.2024.01.005 38301742 PMC12217443

[8] Sargeant JA , Henson J , King JA , Yates T , Khunti K , Davies MJ . A review of the effects of glucagon‐like peptide‐1 receptor agonists and sodium‐glucose cotransporter 2 inhibitors on lean body mass in humans. Endocrinol Metab (Seoul) 2019;34:247–262. 10.3803/EnM.2019.34.3.247 31565876 PMC6769337

[9] Wilding JPH , Batterham RL , Calanna S , Davies M , Van Gaal LF , Lingvay I , *et al*.; STEP 1 Study Group . Once‐weekly semaglutide in adults with overweight or obesity. N Engl J Med 2021;384:989–1002. 10.1056/NEJMoa2032183 33567185

[10] Zainul O , Perry D , Pan M , Lau J , Zarzuela K , Kim R , *et al*. Malnutrition in heart failure with preserved ejection fraction. J Am Geriatr Soc 2023;71:3367–3375. 10.1111/jgs.18590 37706670 PMC10753516

[11] Pandey A , Kitzman D , Reeves G . Frailty is intertwined with heart failure: Mechanisms, prevalence, prognosis, assessment, and management. JACC Heart Fail 2019;7:1001–1011. 10.1016/j.jchf.2019.10.005 31779921 PMC7098068

[12] Pandey A , Kitzman D , Whellan DJ , Duncan PW , Mentz RJ , Pastva AM , *et al*. Frailty among older decompensated heart failure patients: Prevalence, association with patient‐centered outcomes, and efficient detection methods. JACC Heart Fail 2019;7:1079–1088. 10.1016/j.jchf.2019.10.003 31779931 PMC8067953

[13] Hansen MS , Wolfel EM , Jeromdesella S , Moller JK , Ejersted C , Jorgensen NR , *et al*. Once‐weekly semaglutide versus placebo in adults with increased fracture risk: A randomised, double‐blinded, two‐centre, phase 2 trial. EClinicalMedicine 2024;72:102624. 10.1016/j.eclinm.2024.102624 38737002 PMC11087719

